# Perfil clinicoepidemiológico de la infección por Chikungunya en casos hospitalarios atendidos en 2015 en Mérida, México

**DOI:** 10.26633/RPSP.2017.91

**Published:** 2017-06-19

**Authors:** Nina Méndez, Luis Baeza-Herrera, Rafael Ojeda-Baranda, Oswaldo Huchim-Lara, Salvador Gómez-Carro

**Affiliations:** 1 Universidad Marista de Mérida Universidad Marista de Mérida Mérida, Yucatán México Universidad Marista de Mérida, Mérida, Yucatán, México.; 2 Hospital Regional de Alta Especialidad de la Península de Yucatán Hospital Regional de Alta Especialidad de la Península de Yucatán Mérida, Yucatán México Hospital Regional de Alta Especialidad de la Península de Yucatán, Mérida, Yucatán, México.; 3 Hospital General “Agustín O’Horán”, Servicios de Salud de Yucatán Unidad de Vigilancia Epidemiológica Mérida, Yucatán México Hospital General “Agustín O’Horán”, Servicios de Salud de Yucatán, Unidad de Vigilancia Epidemiológica, Mérida, Yucatán, México.

**Keywords:** Fiebre chikungunya, Aedes aegypti, signos y síntomas, vigilancia epidemiológica, México, Chikungunya fever, Aedes aegypti, signs and symptoms, epidemiological surveillance, México, Febre de chikungunya, Aedes aegypti, sinais e sintomas, vigilância epidemiológica, Mexico

## Abstract

**Objetivos.:**

*Describir el perfil epidemiológico y analizar las manifestaciones clínicas por grupo de edad de los casos de chikungunya atendidos en 2015 en un hospital general de Mérida, México*.

**Métodos.:**

*Estudio observacional en el cual se describen los casos de Chikungunya registrados por el Departamento de Vigilancia Epidemiológica entre julio y diciembre de 2015 y análisis de las manifestaciones clínicas porgrupo de edad. La asociación entre los signos y síntomas y el ingreso hospitalario se analizó mediante un modelo de regresión logística*.

**Resultados.:**

*En total se atendieron 830 casos diagnosticados de Chikungunya. La media de edad fue 27,4 ± 17,05 años y la razón de sexos, 1:1,4. Los casos procedieron en su mayoría del medio urbano (n = 592, 87%) y, en particular, de la ciudad capital. En 723 (90%) casos se notificó haber tenido contacto con el vector artrópodo en el peridomicilio. Las alteraciones musculares y articulares fueron más frecuentes en los grupos de mayor edad, mientras que el exantema lo fue entre los más jóvenes. Las manifestaciones de gravedad neurológicas y no neurológicas no tuvieron predominio por edad*.

**Conclusiones.:**

*Los casos de Chikungunya atendidos fueron mayormente cuadros benignos autolimitados, mientras que las manifestaciones neurológicas como letargia, vómitos persistentes, hemorragia, fotofobia y cefalea intensa fueron predictores de la gravedad y su identificación oportuna en los menores de un año de edad fue posible mediante la vigilancia estrecha*.

## Abstract (en)

**Objectives.:**

Describe the epidemiological profile and analyze clinical manifestations by age group in chikungunya patients treated in 2015 in a Mérida, Mexico general hospital.

**Methods.:**

An observational study describing chikungunya cases recorded by the Department of Epidemiological Surveillance from July to December 2015 and analyzing clinical manifestations by age group. A logistic regression model was used to analyze the association between signs and symptoms and hospital admission.

**Results.:**

A total of 830 diagnosed chikungunya cases were treated. Average age was 27.4 ± 17.05 years and the male-to-female ratio was 1:1.4. Most patients came from urban areas (n = 592, 87%) and, in particular, the capital city. Contact with the arthropod vector in the peridomiciliary area was reported in 723 (90%) cases. Muscular and joint impairments were more frequent in the older groups, while rash was more frequent in younger groups. Neurological and non-neurological manifestations of severity did not show greater prevalence in any one age group.

**Conclusions.:**

Chikungunya patients had primarily self-limited benign cases, while neurological manifestations such as lethargy, persistent vomiting, hemorrhage, photophobia, and intense headache were predictive of severity and their timely identification in children under 1 year of age was possible through close monitoring.

## RESUMO

**Objetivos.:**

Descrever o perfil epidemiológico e analisar as manifestações clínicas por faixa etária dos casos de chikungunya atendidos em 2015 em um hospital geral de Mérida, México.

**Métodos.:**

Estudo observacional em que são descritos os casos de chikungunya registrados pelo departamento de vigilância epidemiológica entre julho e dezembro de 2015 e é feita uma análise das manifestações clínicas por faixa etária. A associação entre os sinais e sintomas e a admissão hospitalar foi analisada em um modelo de regressão logística.

**Resultados.:**

Ao todo, foram atendidos 830 casos diagnosticados de chikungunya. A idade média foi 27,4 ± 17,05 anos e a proporção entre os sexos foi de 1:1,4. Os casos foram procedentes na sua maioria da zona urbana (n = 592, 87%) e, em particular, da capital. Em 723 casos (90%) foi notificado o contato peridomiciliar com o vetor artrópode. Manifestações musculares e articulares foram mais frequentes nas faixas etárias avançadas, enquanto que o exantema foi mais comum em jovens. As manifestações neurológicas e não neurológicas graves não apresentaram predomínio por idade.

**Conclusões.:**

Os casos de chikungunya atendidos foram na sua maioria quadros benignos autolimitados, enquanto que as manifestações neurológicas como letargia, vômitos persistentes, hemorragia, fotofobia e cefaleia intensa foram preditivas da gravidade da doença e a identificação oportuna em crianças menores de 1 ano foi possível com a vigilância rigorosa.

El Chikungunya es un virus que produce la enfermedad febril homónima y se transmite mediante los vectores artrópodos del género Aedes. En el estado de Yucatán, *Aedes aegypti* es su principal vector transmisor (1).

Existe evidencia histórica puesta de manifiesto por Halstead que indica que la infección por chikungunya no es una enfermedad emergente en la Región de las Américas, sino reemergente. Sus orígenes se remontan a finales de la década de 1820 y su reingreso en la Región en 2013 marcó el inicio de los brotes en las zonas donde se encuentra del vector transmisor (2, 3).

Antes de su regreso a la Región, el virus chikungunya causó el brote en Kenia de 2004 y, posteriormente, alcanzó numerosas islas del Pacífico, entre ellas la de Reunión, donde se notificaron 46 000 casos y 284 muertes. En 2006, se notificaron casos importados de esta infección en Europa (1).

El primer caso de transmisión local en la Región se notificó en las islas de Saint Martin y Martinica en 2013. En 2014, se notificaron casos en otras islas del Caribe, Centro América y Sudamérica y en ese mismo año se confirmó el primer caso importado en México. Hasta la semana epidemiológica número 45 de 2014, en el estado de Chiapas se habían notificado 11 casos importados y 14 autóctonos. Luego se notificaron en otros estados de México. En el estado de Yucatán, los dos primeros casos se confirmaron en mayo de 2015 y el pico de máxima incidencia se observó en septiembre de 2015 (4, 5). Durante 2015, hubo12 588 casos confirmados de fiebre por Chikungunya en México. De ellos, 1 669 ocurrieron en el estado de Yucatán y 1 falleció (6-8).

La enfermedad causada por el virus Chikungunya suele manifestarse clínicamente entre los tres y siete días después de la exposición y la picadura del vector transmisor. El inicio de la fase aguda debuta con dolor y fiebre y se considera diagnóstica cuando, además, se acompaña de dos o más de los siguientes signos o síntomas: cefalea, dolor de espalda, que suele ser característico de la enfermedad, mialgias, náuseas, vómitos, exantema o conjuntivitis.

El dolor y la inflamación afectan de manera característica a las articulaciones de las manos y de los tobillos, aunque también pueden afectar a las articulaciones de mayor tamaño. En 40-50% de los casos se ha encontrado exantema maculopapular en el tórax y edema facial. La persistencia de las artralgias durante cuatro meses se ha notificado en 33% de los pacientes infectados. El tiempo medio en que los pacientes afectados por la artralgia acuden en busca de tratamiento especializado por reumatología es de 8 meses. Por otro lado, las pruebas disponibles sugieren que la infección puede cursar con presentación subclínica, que se ha asociado con algunos rasgos genéticos (9-11).

Conocer las características epidemiológicas y las manifestaciones clínicas de los pacientes que padecieron la infección por Chikungunya en la zona endémica de Yucatán puede ayudar a identificar las áreas de mejora diagnóstica, comprender mejor la transmisión e identificar la presencia o ausencia de grupos particularmente vulnerables. Por ello, el objetivo de este estudio es describir el perfil epidemiológico y analizar las manifestaciones clínicas por grupos de edad de los casos de Chikungunya atendidos en 2015 en un hospital general de Mérida, México.

## MATERIALES Y MÉTODOS

Se realizó un estudio descriptivo en el cual se describieron los casos de infección por Chikungunya del Departamento de vigilancia epidemiológica hospitalario del Hospital General O’Horan entre julio y diciembre de 2015.

En las directrices diagnósticas del Instituto de Referencia Epidemiológica de México se define como caso confirmado de Chikungunya todo aquel que cuente con un resultado positivo de laboratorio, ya sea por RT PCR durante los primeros cinco días del inicio de la fiebre o, mediante la identificación de anticuerpos IgM, a partir del sexto día del inicio de la fiebre. Además, se definen como casos de Chikungunya los que muestran asociación epidemiológica con casos confirmados en la localidad. En el algoritmo diagnóstico vigente a partir de junio 2015 se estipuló que, al haberse confirmado la circulación del virus en el estado de Yucatán, únicamente se procesaría 5% de las muestras de los casos sospechosos y de todos los casos atendidos en el hospital en recién nacidos y lactantes con factores de riesgo.

A los efectos del presente estudio, se consideró criterio de inclusión de casos el que éstos fuesen atendidos en un hospital y estuvieran registrados en el departamento de vigilancia epidemiológica del hospital público general localizado en la capital del estado de Yucatán, México. Se excluyeron los casos que se descartaron con las pruebas de laboratorio.

Las variables del estudio fueron, primero, el medio rural o urbano y el hábito de movilidad**. Esta variable se define como dicotómica si el paciente habita en medio rural o urbano y como categórica, si éste considera que se infectó en el domicilio, en el trabajo o en la escuela. Segundo, el ingreso hospitalario**. Aunque todos los casos de este estudio se atendieron en el hospital, durante el tiempo en que se recolectó la información se encontraba habilitado un espacio hospitalario para servir de *triage* de los casos de dengue y de chikungunya, de manera que después de una primera valoración médica y de la búsqueda de factores de riesgo, sobre la base de los resultados y de las manifestaciones clínicas, el paciente podía permanecer en el hospital tras el ingreso o irse para ser atendido de forma ambulatoria. Tercero, las manifestaciones clínicas, una variable dicotómica definida como la presencia o la ausencia de cefalea, mialgias, poliartralgias, sensación de rigidez en las articulaciones, artritis e inflamación visible, lumbalgia, exantema, prurito, náuseas, vómitos, escalofríos, fotofobia y estupor. Y cuarto, los grupos de edad, con la cual las manifestaciones clínicas identificadas en los casos atendidos se presentan en categorías de edad a partir del grupo de 5-14 años. En los grupos de < 1 y de 1-4 años no se tabula la sintomatología debido a la dificultad para establecer su presencia en pacientes de dichas edades.

Los datos se registraron en una base de datos en la Unidad de Vigilancia Epidemiológica Hospitalaria anonimizada y codificada para ser analizada posteriormente por los investigadores. Para las variables categóricas se estimaron proporciones y para las continuas, las medias y desviaciones estándar. Asimismo, las estimaciones se acompañaron de sus correspondientes intervalos de confianza de 95%. Para averiguar si existía una asociación entre las manifestaciones clínicas y el ingreso hospitalario en los pacientes ≥ 5 años se construyó un modelo de regresión logística dicotómica. La bondad de ajuste del modelo se valoró mediante la prueba Hosmer-Lomeshow. Se omitieron las manifestaciones clínicas no significativas a un valor alfa < 0,05. Los análisis estadísticos se realizaron con STATA v.12.

## RESULTADOS

Entre julio y diciembre de 2015 se atendió a un total de 830 casos diagnosticados de chikungunya. La media de edad (y la desviación estándar) fue 27,4 ± 17,0 años, y la razón de sexos, 1:1,4. Se excluyeron 16 pacientes tras haber descartado que padecían una infección por chikungunya y 13 por alta voluntaria o traslado a otras unidades médicas. Entre los casos descartados se diagnosticaron nueve positivos a dengue, tres a enterovirus, dos a ricketsiosis, una infección por virus Newcastle y una por toxoplasma.

Los casos procedieron en su mayoría del medio urbano (592, 87%) y, en particular, de la ciudad capital. Sin embargo, también se atendieron 32 pacientes de otros estados de México y 13 viajeros extranjeros que adquirieron el virus durante su estancia.

En cuanto a los hábitos de movilidad de los pacientes, en 723 (90%) casos se notificó que habían tenido contacto con el vector artrópodo dentro del hogar o en sus alrededores, mientras que 10% restante recordó haber tenido contacto con los moscos en el trabajo, la escuela u otros lugares visitados.

Respecto a la media de días transcurridos entre el inicio de los síntomas y la primera atención médica se observó un aumento progresivo con la edad: fue menor en los niños (media = 2,22 ± 0,12; IC95%: 1,97-2,46) que en los adul-tos (2,31 ± 0,33; IC95%: 2,25-2,38) y los adultos mayores (2,87 ± 0,11; IC95%:.2,65-3,10).

De los 803 pacientes atendidos, 672 (83,7%) recibieron atención ambulatoria y no se registraron complicaciones en sus visitas subsecuentes, mientras los 131 restantes fueron ingresados en los servicios hospitalarios correspondientes según su edad y casuística. El único fallecimiento se produjo en el grupo de edad de < 1 año. De dichos 803 pacientes, 570 (71%) fueron enviados directamente al hospital sin previa valoración desde las unidades de primer contacto, y 233 (29%) llegaron al hospital con una valoración médica previa. En el cuadro 1 se presenta la distribución según el tipo de atención recibida (hospitalaria o ambulatoria), la edad y el sexo de los 803 casos Chikungunya.

Las manifestaciones clínicas observadas en los 48 pacientes < 1 año hospitalizados fueron: fiebre (media 39,1°C), llanto incoercible de duración variable e irritabilidad. En 20 de ellos (34,4%) se produjo intolerancia a la vía oral, 4 (6,9%) desarrollaron adenomegalias y se apreciaron adenomegalias, y 2 también presentaron hepatomegalia y esplenomegalia. Las lesiones dérmicas en este grupo de edad fueron inespecíficas, pero al menos 4 (6,9%) desarrollaron lesiones maculares eritematosas únicas en miembros inferiores.

En el grupo de 1-4 años de edad la media de la temperatura corporal fue 39° C durante la fase aguda y las manifestaciones clínicas fueron dolor inespecífico, adinamia y pérdida del apetito en todos los casos. En este grupo de edad, 24 (63,1%) niños tenían eritema maculopapular difuso y los 16 casos que fueron hospitalizados tuvieron letargia.

Las manifestaciones más frecuentemente observadas entre los pacientes ≥ 5 años se presentan en el cuadro 2. Las alteraciones musculares y articulares fueron más comunes en los grupos de mayor edad, mientras que el exantema, por el contrario, fue más frecuente entre los más jóvenes. En la aparición de signos y síntomas como náuseas, vómitos, escalofríos, fotofobia y letargia no se apreció predominio por grupo de edad.

**CUADRO 1.** Distribución según el tipo de atención recibida, la edad y el sexo de los 803 casos Chikungunya, Mérida, México, 2015

Edad (años)	Tipo de atencion recibida				
	Hospitalaria		Ambulatoria		
	Hombres	Mujeres	Hombres	Mujeres	
<1	28	20	23	16	87
1-4	9	7	15	7	38
5-14	12	10	38	53	113
15-24	11	10	75	105	201
25-44	4	14	72	137	227
45-64	4	1	36	78	119
≥65	1	0	5	12	18
Total	69	62	264	408	803

En el modelo logístico se incluyeron cinco variables, de las cuales la letargia, la fotofobia y la hemorragia se asociaron positivamente con el ingreso hospitalario, la variable dependiente, mientras que el vómito persistente se asoció negativamente al igual que la cefalea intensa, aunque no se puede descartar la ausencia de asociación con el ingreso hospitalario en esta última variable, habida cuenta de su IC95%. La bondad de ajuste del modelo fue 4,39 (p = 0,495) (pseudo r^2^ = 0,15) (cuadro 3).

Las manifestaciones clínicas infrecuentes observadas (en menos de 3 casos) en los pacientes ≥ 25 años que fueron predictores estadísticos perfectos del ingreso hospitalario se reducen a la presencia de alteraciones neurológicas, incluidos el estupor y la arreflexia, y de manifestaciones no neurológicas, entre las cuales se identificaron el dolor abdominal intenso, la disnea y la ictericia.

## DISCUSIÓN

En este artículo se han descrito los casos de Chikungunya atendidos en hospitales de Yucatán. Se ha observado que en sumayoría los casos procedían del medio urbano (y principalmente de la ciudad capital) y refirieron haber tenido contacto con el vector en el domicilio. Las manifestaciones clínicas de los pacientes variaron según la edad. Los casos que fueron hospitalizados difirieron de los que recibieron atención ambulatoria por presentar signos o síntomas que sugirieron mayor gravedad de su cuadro clínico.

**CUADRO 2.** Datos clínicos de los 803 casos de Chikungunya hospitalizados en Mérida, México, 2015

Grupo de edad (n)	Medida	Cefalea	Mialgias	Rigidez	Artralgias	Artritis	Lumbalgia	Exantema	Prurito	Náusea	Vómitos	Escalofríos	Fotofobia	Letargia
5-14	No.	100	103	94	88	57	49	76	71	24	40	53	2	2
(113)	%	88,5	91,2	83,2	77,9	50,4	43,4	67,3	62,8	21,2	35,4	46,9	1,8	1,8
15-24	No.	188	192	169	166	133	134	135	124	51	75	102	8	3
(201)	%	93,5	95,5	84,1	82,6	66,2	66,7	67,2	61,7	25,4	37,3	50,7	4,0	1,5
25-44	No.	214	222	206	198	148	150	152	139	61	80	132	14	3
(227)	%	94,3	97,8	90,7	87,2	65,2	66,1	67,0	61,2	26,9	35,2	58,1	6,2	1,3
45-64	No.	113	117	104	106	85	79	65	53	20	39	66	21	2
(119)	%	95,0	98,3	87,4	89,1	71,4	66,4	54,6	44,5	16,8	32,8	55,5	17,6	1,7
≥ 65	No.	18	18	17	18	14	13	9	8	4	7	9	3	1
(18)	%	100,0	100,0	94,4	100,0	77,8	72,2	50,0	44,4	22,2	38,9	50,0	16,7	5,6
Total	No.	633	652	590	576	437	425	438	388	160	241	362	48	1
	%	78,8	81,2	73,5	71,7	54,4	52,9	54,5	48,3	19,9	30,0	45,1	6,0	0,1

**CUADRO 3.** Regresión logística para analizar la asociación entre el ingreso hospitalario y la presencia de manifestaciones clínicas en los pacientes con Chikungunya, Mérida, México, 2015

Manifestación clínica	Odds Ratio	IC95%	
Letargia	2,70	1,40	5,22
vómito persistente	0,42	0,27	0,67
Hemorragia	1,74	1,12	2,68
Fotofobia	2,53	1,43	4,47
Cefalea intensa	0,19	1.08	0.17
Constante	0,85	0,50	1,44

IC95%: intervalo de confianza de 95%.

Chikungunya es un virus que, al igual que el del dengue y la fiebre amarilla, inició su ciclo de transmisión urbana a partir de la amplificación humana y la presencia de mosquitos en el peridomicilio (12). El predominio de casos urbanos podría explicarse por el hecho de que, en Yucatán, casi la mitad de la población del estado se concentra en la capital, donde es mayor la densidad de población, lo que favorece la transmisión del virus (13). También se observó que el contacto con los mosquitos vectores se produjo mayormente en las inmediaciones de los domicilios o en su interior, lo que sugiere que las medidas de saneamiento del medio podrían no ser óptimas.

La dispersión de Chikungunya pone de manifiesto que es necesario mantener continuamente las medidas de vigilancia vectorial activa, así como la importancia de difundir las estrategias de autocuidado individual y del peridomicilio, para poder prever oportunamente futuros brotes y actuar con eficacia ante nuevos virus transmitidos por el mismo vector (14).

Si bien los resultados de este estudio sugieren que los pacientes hospitalizados fueron primordialmente aquellos que de forma justificada requerían vigilancia estrecha por la gravedad de su cuadro clínico o sus factores de riesgo individual, el hecho de que 83% de ellos sólo necesitara atención ambulatoria también apunta a que, si se hubiese optimizado la atención en los centros de salud de primer contacto, los pacientes habrían podido atenderse desde su unidad médica más cercana sin necesidad de ser referidos a un hospital general, tal como se ha realizado en ciertos lugares donde ha habido brotes (15).

Al analizar los casos de este estudio por grupos de edad, se aprecia que hay variaciones en la presentación clínica de Chikungunya: mayor propensión a las afectaciones musculoarticulares de los pacientes de mayor edad y menor propensión de las manifestaciones dermatológicas en ellos. Aunque las manifestaciones clínicas de gravedad fueron las mismas en los diferentes grupos de edad, no es fácil identificar sobre todo las de tipo neurológico en menores de 1 año, y por ello la vigilancia en este grupo de edad es de vital importancia para evitar complicaciones que pongan en riesgo su vida, como las notificadas durante la epidemia de la isla Reunión (16, 17).

La experiencia basada en el estudio de los brotes de Chikungunya ocurridos en diversas regiones sugiere que en el futuro será importante revisar la evolución de los casos para tener más información sobre sus implicaciones a mediano y largo plazo.

Como conclusión cabe afirmar que los casos de Chikungunya atendidos fueron mayormente cuadros benignos autolimitados. En los pacientes con letargia, fotofobia y hemorragia el riesgo de ingreso hospitalario fue mayor que el de los pacientes sin estos signos y síntomas, y en los que presentaron vómitos persistentes y cefalea intensa el riesgo fue menor que el de los que no lo presentaron. En los niños menores de un año se pudo identificar tempranamente la gravedad mediante su vigilancia clínica estrecha.

### Financiación.

El presente estudio no ha sido financiado.

### Declaración.

Las opiniones expresadas por los autores son de su exclusiva responsabilidad y no reflejan necesariamente los criterios ni la política de la Organización Panamericana de la Salud o de la *RPSP/PAJPH*
